# PlsX and PlsY: Additional roles beyond glycerophospholipid synthesis in Gram-negative bacteria

**DOI:** 10.1128/mbio.02969-24

**Published:** 2024-10-30

**Authors:** Audrey N. Rex, Brent W. Simpson, Gregory Bokinsky, M. Stephen Trent

**Affiliations:** 1 Department of Microbiology, College of Art and Sciences; University of Georgia, Athens, Georgia, USA; 2 Department of Infectious Diseases, College of Veterinary Medicine, University of Georgia, Athens, Georgia, USA; 3 Department of Bionanoscience, Kavli Institute of Nanoscience, Delft University of Technology, Delft, Netherlands; Fred Hutchinson Cancer Center, Seattle, Washington, USA

**Keywords:** PlsX, PlsY, PlsB, glycerophospholipids, phospholipids, phosphatidic acid, Gram-negative bacteria

## Abstract

The unique asymmetry of the Gram-negative outer membrane, with glycerophospholipids (GPLs) in the inner leaflet and lipopolysaccharide (LPS) in the outer leaflet, works to resist external stressors and prevent the entry of toxic compounds. Thus, GPL and LPS synthesis must be tightly controlled to maintain the integrity of this essential structure. We sought to decipher why organisms like *Escherichia coli* possess two redundant pathways—PlsB and PlsX/Y—for synthesis of the GPL precursor lysophosphatidic acid (LPA). LPA is then converted by PlsC to the universal precursor for GPL synthesis, phosphatidic acid (PA). PlsB and PlsC are essential in *E. coli*, indicating they serve as the major pathway for PA synthesis. While loss of PlsX or PlsY individually has little consequence on the cell, the absence of both was lethal. To understand the synthetic lethality of this seemingly redundant PlsX/Y pathway, we performed a suppressor screen. Suppressor analysis indicated that ∆*plsXY* requires increased levels of glycerol-3-phosphate (G3P), a GPL precursor. In agreement, ∆*plsXY* required supplementation with G3P for survival. Furthermore, loss of PlsX dysregulated fatty acid synthesis, resulting in increased long-chain fatty acids. We show that although PlsX/Y together contribute to PA synthesis, they also contribute to the regulation of overall membrane biogenesis. Thus, synthetic lethality of ∆*plsXY* is multifactorial, suggesting that PlsX/Y has been maintained as a redundant system to fine-tune the synthesis of major lipids and promote cell envelope homeostasis.

**IMPORTANCE:**

Gram-negative bacteria must maintain optimal ratios of glycerophospholipids and lipopolysaccharide within the cell envelope for viability. Maintenance of proper outer membrane asymmetry allows for resistance to toxins and antibiotics. Here, we describe additional roles of PlsX and PlsY in *Escherichia coli* beyond lysophosphatidic acid synthesis, a key precursor of all glycerophospholipids. These findings suggest that PlsX and PlsY also play a larger role in impacting homeostasis of lipid synthesis.

## INTRODUCTION

The bacterial cell envelope has a complex composition that serves to provide protection from various environmental stresses while allowing for influx of nutrients (1). Gram-positive bacteria are monoderms with a single lipid bilayer and thick peptidoglycan cell wall. In contrast, Gram-negative bacteria are diderms with two lipid bilayers encasing a thin layer of peptidoglycan. The Gram-negative inner membrane is symmetric with both leaflets composed of glycerophospholipids (GPLs). The outer membrane (OM), however, is asymmetric with the inner leaflet consisting of GPLs and the outer leaflet containing the glycolipid lipopolysaccharide (LPS) (2, 3). Asymmetry of the OM provides a robust barrier to protect the cell from toxic molecules and antibiotics, given that proper lipid composition is maintained. Currently, antibiotic resistance represents a major global health crisis as bacteria have evolved resistance to a broad range of treatments (4). Furthermore, the unique properties of the Gram-negative cell envelope make these organisms naturally resistant to many antibiotics that are otherwise effective against Gram-positive bacteria. Thus, understanding assembly and maintenance of the OM is crucial for developing targeted antimicrobial treatments.

**Fig 1:**
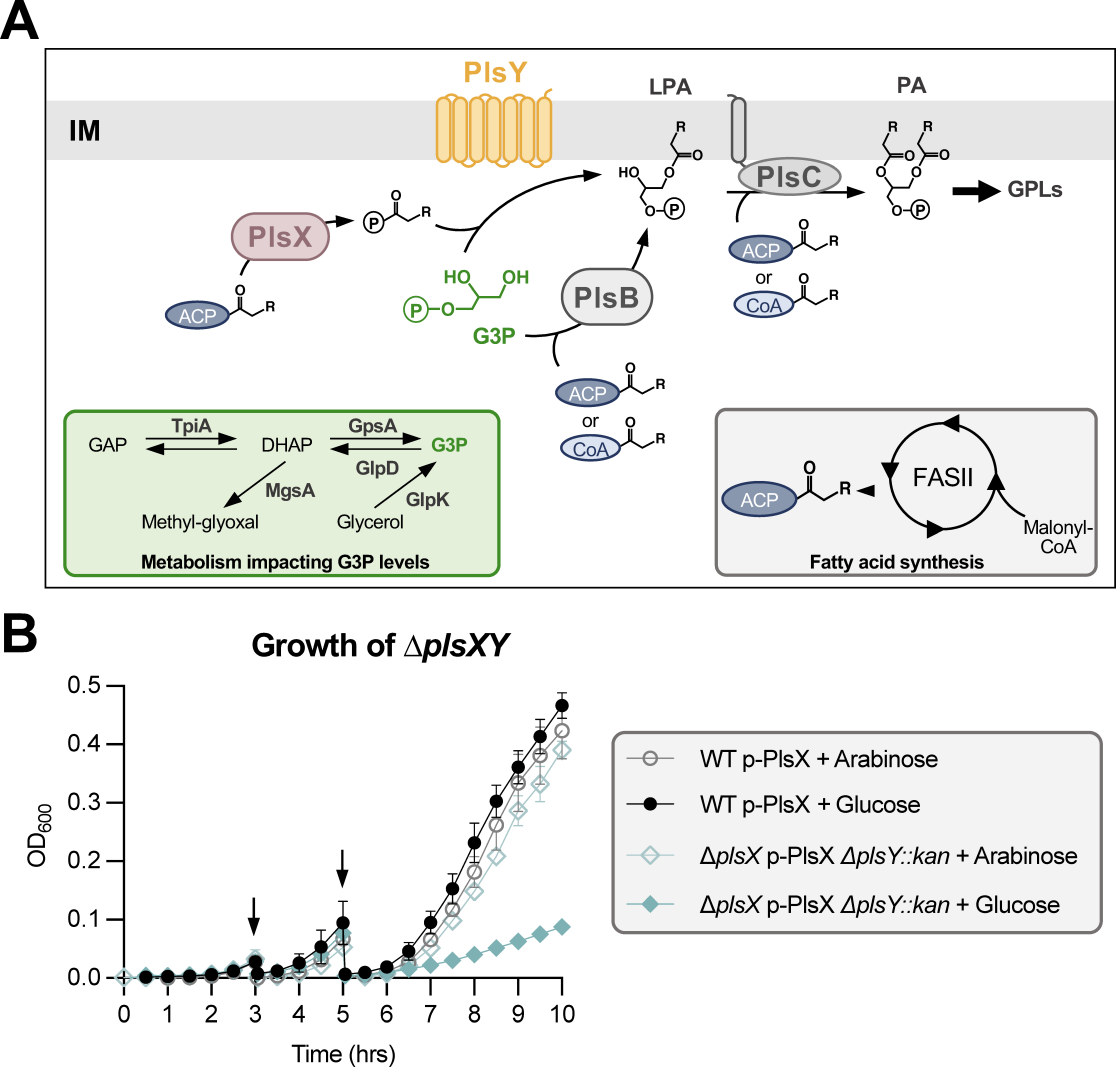
Loss of PlsX and PlsY results in a synthetically sick strain. (**A**) *Escherichia coli* synthesizes PA via a two-step acylation of G3P. G3P substrate is generated either via TpiA/GpsA, first generating dihydroxyacetone (DHAP) from glyceraldehyde-3-phosphate (GAP) before conversion to G3P, or through *de-novo* synthesis via GlpK. G3P can be reversed to DHAP via GlpD. DHAP can be shunted irreversibly to methyl-glyoxal, a toxic compound, via MgsA (green inset). Fatty Acid Synthesis (FASII) generates acyl-ACP of appropriate carbon lengths to serve as acyl donors (gray inset). The first acylation of G3P is catalyzed by either PlsB or PlsY, enzymes that produce LPA. The second acylation step generates PA via the universally conserved protein, PlsC. PlsB and PlsC use both acyl-ACP and acyl-CoA as donor substrates, whereas PlsY can only use an acyl-PO_4_ that is specifically generated by PlsX. PA is immediately used in GPL biosynthesis. (**B**) Depletion growth curve showing a synthetic growth defect of ∆*plsX* ∆*plsY*. Strains were grown in lysogeny broth (LB) amp under inducing (0.05% arabinose) or repressing (0.05% glucose) conditions. Two back-dilutions (denoted by arrows) were performed at three and five hours to sufficiently deplete PlsX. Error bars represent standard deviation (SD) from three biological replicates and are not shown if smaller than the symbols.

The universal precursor for GPL synthesis is phosphatidic acid (PA), which is enzymatically synthesized through a two-step acylation of glycerol-3-phosphate (G3P) (5–9). Bacteria may contain two separate enzymes for the first acylation step of G3P. PlsB (G3P 1-*O*-acyltransferase) synthesizes lysophosphatidic acid (LPA), utilizing either acyl-acyl carrier protein (ACP) or acyl-coenzyme A (CoA) as the fatty acyl donor (Fig. 1A). Alternatively, LPA can be generated by PlsY (G3P 1-*O*-acyltransferase) which requires an acyl-phosphate (PO_4_) as a specialized acyl donor. The latter is synthesized by PlsX through the conversion of acyl-ACP to acyl-PO_4_. Next, PlsC (LPA 2-*O*-acyltransferase) adds an additional acyl chain to LPA and, like PlsB, can utilize acyl-ACP and/or acyl-CoA (8, 9) (Fig. 1A). Although discovered after PlsB, the most widespread mechanism for the first acylation reaction and generation of LPA is through PlsX/Y. In fact, most Gram-negative proteobacteria, especially occurring in γ-proteobacteria like *E. coli*, maintain both systems—PlsX/Y and PlsB—for the initiation of PA synthesis (10). It should be noted that the only bacterial species known to maintain only PlsB/C is Xanthomonadales, a subset of γ-proteobacteria (8).

In species with only one route for PA synthesis, either through PlsB/C or PlsX/Y/C, each enzyme is essential for growth. Therefore, in *E. coli* it would be expected that PlsB, PlsX, or PlsY would not be essential due to redundancy within this pathway. However, *plsX* and *plsY* single mutants are viable while PlsB is essential; these results suggest that PlsB activity is the major enzymatic route for the generation of LPA for GPL synthesis (11). Surprisingly, loss of both PlsX and PlsY is synthetically lethal in *E. coli* (12). Since the enzymatic function of PlsY is directly dependent upon a donor generated by PlsX and the fact that single *plsX* and *plsY* mutants are viable yet the double mutant is not, it suggests that PlsX and PlsY have an additional role outside of the traditional synthetic pathway.

Here, we sought to dissect the roles of PlsX and PlsY in γ-proteobacteria by selecting for suppressors of the synthetic lethal phenotype of a *plsXY* double mutant. Characterization of suppressor mutations suggested that ∆*plsXY* had insufficient precursors to support GPL synthesis. We found that loss of PlsX results in an increased concentration of very long-chain (C18:0/1-C20:0/1) acyl-ACPs. Long-chain acyl-ACPs are sensed by the cell to decrease initiation of fatty acid synthesis (FASII in *E. coli*) (13), which was likely part of the toxicity from loss of PlsX and PlsY. These data suggested that PlsX had a regulatory role in balancing GPL precursors. In agreement, slowing down FASII through chemical treatment was toxic to ∆*plsY*, mimicking the synthetic lethality of the *plsXY* mutant. Together, our data suggest that the combination of reduced PA flux, imbalance of GPL precursors caused by the loss of PlsX, and a tertiary role of PlsY results in synthetic lethality of ∆*plsXY*.

## RESULTS

### Loss of both PlsX and PlsY is synthetically lethal

PA is the universal precursor for GPL synthesis. *E. coli* and other proteobacteria have evolved to maintain two mechanisms for the first step of PA synthesis: PlsB, which is essential, and PlsX and PlsY, which are not individually essential (Fig. 1A). In 2007, synthetic lethality of a *plsXY* double mutant was suggested by the inability to generate colonies via phage transduction (12). However, this method is not the most robust way to prove synthetic lethality. In this new era of sequencing, we can quickly, accurately, and affordably identify suppressors across the genome of an entire strain, so we sought first to validate this synthetic lethality and to then select for suppressors. As expected, in single mutants lacking *plsX* or *plsY*, there was no apparent growth defect (Fig. S1). Next, we introduced a *∆plsY::kan* allele into the *plsX* mutant while expressing *plsX in trans* from an arabinose-inducible promoter. These cells were grown under inducing (+ arabinose) conditions overnight, washed, and switched to repressing (+ glucose) conditions. As expected, the *plsXY* double mutant displayed a severe synthetic growth defect after two dilutions to sufficiently deplete PlsX levels, supporting synthetic lethality (Fig. 1B).

To fully demonstrate that ∆*plsXY* is synthetically lethal, we performed co-transduction using P1 phage raised on ∆*plsY* also containing a kanamycin resistance cassette in *ygjH* (*∆ygjH::kan*), a gene closely linked to *plsY*. Following P1 transduction into a ∆*plsX* recipient strain, bacteria were selected on kanamycin and screened for both gene deletion, via primers that anneal to regions flanking *plsY*, and gene duplication, via primers that anneal to regions within *plsY*. We obtained an experimental co-transduction frequency of 51.3% into our wild-type (WT) strain, W3110. The co-transduction frequency between Δ*ygjH::kan* and Δ*plsY* was disrupted when brought into Δ*plsX* recipient strain with 0% containing the deletion of *plsY* (Table S1). We also tested to see if gene deletion order mattered and found via co-transduction frequencies that it does not (Table S1). Together, these results conclusively demonstrate synthetic lethality of a *plsXY* double mutant, regardless of gene deletion order.

It should be noted that *plsX* is in an operon with essential fatty acid synthesis genes. We conducted RNA-sequencing (RNA-seq) to determine if there were any polar effects on *fabH*, *fabD*, and *fabG* gene expression, as well as to probe for possible dysregulation of other genes outside of this operon. In a ∆*plsX* background, the only changes in gene expression of statistical significance (fold change >2, false discovery rate (FDR) *P*-value ≤ 0.05) were *plsX* transcripts, which were absent due to the gene deletion (−504-fold change, 4.48E-43 FDR *P*-value), and *cspG* (cold shock protein) transcripts (−3.18-fold change, 0.02 FDR *P*-value; Data Set S1). There was no evidence of polar effects on *fabH*, *fabD*, *and fabG* in ∆*plsX* in our working conditions.

### Isolation of suppressor mutations that rescue ∆*plsXY* synthetic lethality

**Fig 2:**
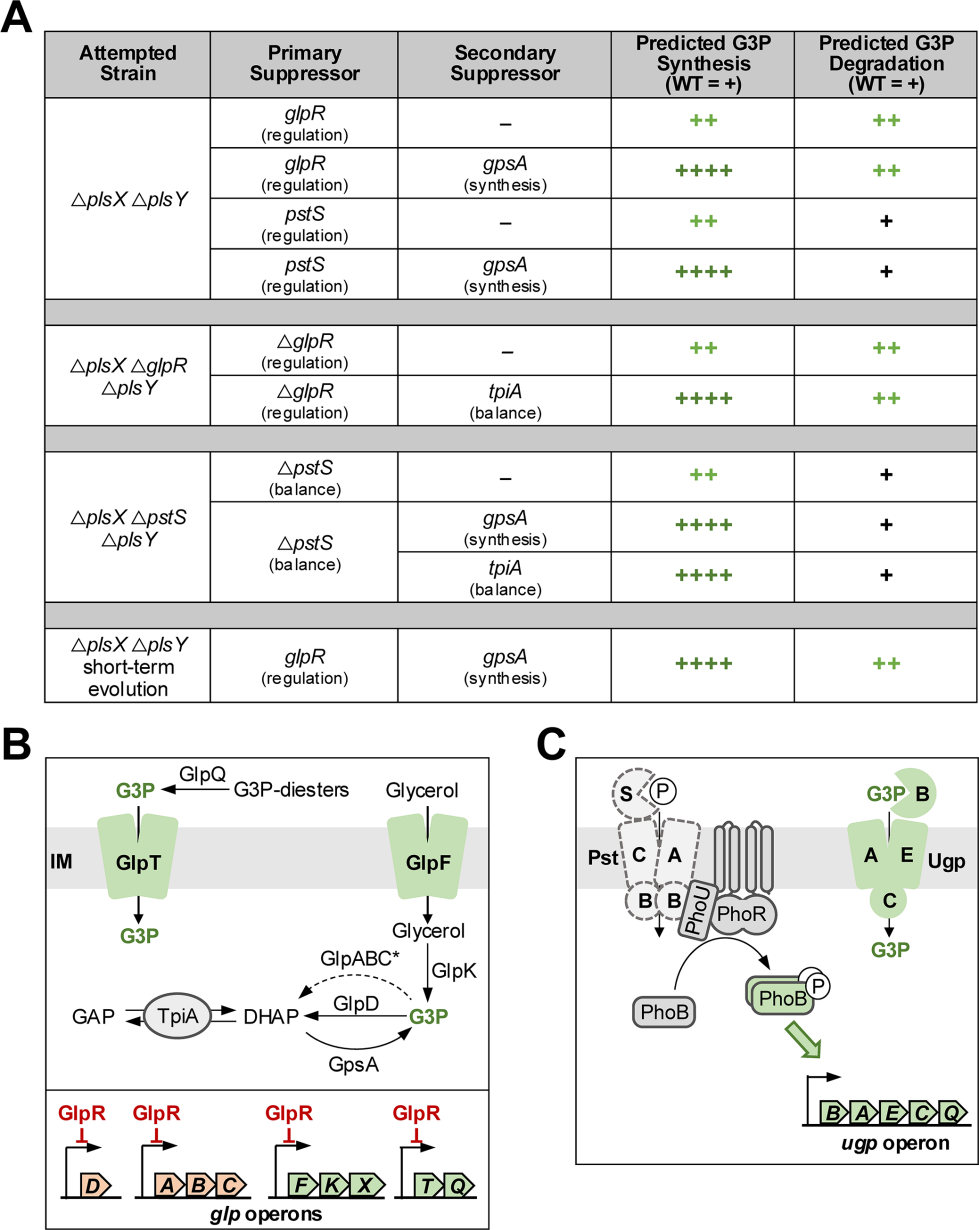
Isolation of suppressor mutations that rescue ∆*plsX* ∆*plsY* synthetic lethality. (**A**) Summary of primary suppressors allowing for the generation of *plsX plsY* double mutants via phage transduction, followed by secondary suppressors that arose after revival from frozen stocks. Outcomes of each suppressor strain are summarized in the rightmost columns regarding predicted changes in G3P concentration. Levels of G3P in WT are shown as “+” to indicate a balance between synthesis and degradation. “Regulation” refers to biological processes that would impact the expression of genes that increase or decrease levels of G3P and substrates for synthesis, while “balance” refers to processes directly involved in synthesis/degradation of G3P. (**B**) Overview of the *glp* regulon—consisting of nine *glp* genes involved in the regulation of G3P—with orange indicating degradation of G3P and green indicating import or synthesis of G3P. GlpR acts as a repressor for all genes, repressing in the presence of glucose and unbound to DNA in the presence of glycerol or G3P. Suppressors resulting in dysregulation in GlpR could result in constitutive activation of the *glp* regulon. * Indicates anaerobic complex. (**C**) Inactivation of the PstSABC complex (represented by dashed lines) results in constitutive activation of the Pho regulon, which includes the *ugpBAECQ* operon. UgpBAECQ is involved in breakdown of glycerophosphodiesters and import of G3P from the periplasmic space.

To identify genes that overcome the synthetic lethal phenotype, we isolated suppressors via large-scale transduction as described previously (14), with the Δ*plsY::kan* allele into a ∆*plsX* recipient strain and vice versa (∆*plsX::kan* allele into a ∆*plsY* recipient strain). Suppressors arose within 16 hours, were isolated, and mutations were identified by whole-genome sequencing (WGS). Mutations arose frequently in the genes encoding GlpR (suppressors 1–6) and PstS (suppressors 7–9; Fig. 2A; Data Set S1). Importantly, both GlpR and PstS impact regulatory metabolic pathways of the same GPL precursor, G3P. GlpR is a repressor of the *glp* regulon, composed of nine genes that help balance G3P levels (Fig. 2B) (15). Suppressor mutations in GlpR ranged from single nucleotide mutations, deletion of base pairs, and insertion of mobile elements. These substantial genetic changes suggested the mutations resulted in loss of GlpR function. The second major suppressor, disruptions of the gene encoding PstS, impacted the PstSABC complex that activates the DNA-binding regulator PhoB in response to the level of environmental inorganic phosphate (Fig. 2C). Inactivation of any protein in the PstSABC complex results in constitutive activation of PhoB-regulated genes (16). PhoB regulates the expression of *ugpBAECQ* which encodes an ABC transporter system for uptake of G3P, UgpAEC, and the G3P periplasmic binding protein UgpB (Fig. 2C). Disruptive mutations in *pstS* (insertion of mobile elements and large deletions) suggested loss of the encoding protein’s function, resulting in constitutive activation of PhoB.

To test if the suppressor mutations were loss-of-function alleles, we generated the double mutants, ∆*plsX* ∆*glpR* and ∆*plsX* ∆*pstS*. We then sought to introduce the Δ*plsY::kan* allele using P1 transduction. The triple mutants were viable, indicating the loss of GlpR or PstS suppressed synthetic lethality. These suppressors suggested that G3P levels must be altered in the *plsXY* double mutant by either removing repression of the *glp* regulon by GlpR or through constitutive activation of PhoB. Notably, initial suppressor mutations that disrupted *glpR* and *pstS* did not result in stably growing strains. Additional colony morphologies appeared upon reviving the bacteria from glycerol stocks. Colonies were re-isolated, re-sequenced, and found to contain secondary suppressors in genes associated with G3P synthesis, including TpiA (suppressors 10–12) and GpsA (suppressor 13; Fig. 2A; Data Set S1). GpsA, a G3P dehydrogenase, directly converts the glycolytic intermediate dihydroxyacetone-phosphate (DHAP) to G3P (7), whereas TpiA acts as an isomerase and balances the cellular levels of glyceraldehyde 3-phosphate (GAP) and DHAP (Fig. 1A inset). These results suggested that loss of *plsXY* required increased levels of G3P, either by pushing synthetic pathways to produce more G3P or to increase its transport. It is likely that the initial *glpR* and *pstS* suppressor mutations were not sufficient to stably increase cytoplasmic G3P levels. Metabolism of G3P is highly tunable through (i) TpiA reversibility and (ii) balance of G3P biosynthesis through GpsA and G3P degradation through GlpD (aerobic) or GlpABC (anaerobic; Fig. 2B) (7, 17). In addition, G3P generation via GlpK (glycerol kinase; Fig. 1A inset) is regulated by a glycolysis intermediate, fructose 1,6-bisphosphate, which is degraded by GlpX (18). It is likely that the secondary mutations were needed to favor one direction in G3P metabolism.

However, GpsA and TpiA both contribute to overall flux of DHAP which can be shunted to a toxic byproduct, methylglyoxal, via MgsA (methylglyoxal synthase; Fig. 1A inset). Toxicity can be overcome in the presence of glutathione, which spontaneously reacts with methylglyoxal (19). To confirm that synthetic lethality of ∆*plsXY* was not due to build-up of methylglyoxal, we aimed to build a ∆*plsX* ∆*mgsA* ∆*plsY::kan* mutant via P1 phage transduction, but we were unable to obtain colonies despite repeated attempts. Additionally, no suppressors arose in genes related to glutathione synthesis. Therefore, synthetic lethality of ∆*plsXY* is not due to growth stasis via methylglyoxal accumulation.

Before investigating how G3P levels were altered, we needed to know how many mutations were necessary to get stable suppression of the synthetic lethality. We turned to a short-term evolution approach to determine what mutations were required to stabilize growth. Every 24 hours, a culture of *∆plsXY* was back-diluted 1:10 into fresh lysogeny broth (LB) media and incubated at 37°C until the mutant reached an equivalent overnight density compared to wild-type W3110. In a pilot experiment, an evolved strain only obtained single nucleotide mutations in two genes even after 26 days of passaging—*glpR* and *gpsA* (suppressor 14; Fig. 2A; Data Set S1). These results indicated that the secondary mutations likely resulted in stable strains. Altogether, our suppressor analysis suggested that cytoplasmic G3P levels must be increased to support the viability of Δ*plsXY*.

### Increasing G3P concentration rescues ∆*plsXY* synthetic lethality

**Fig 3:**
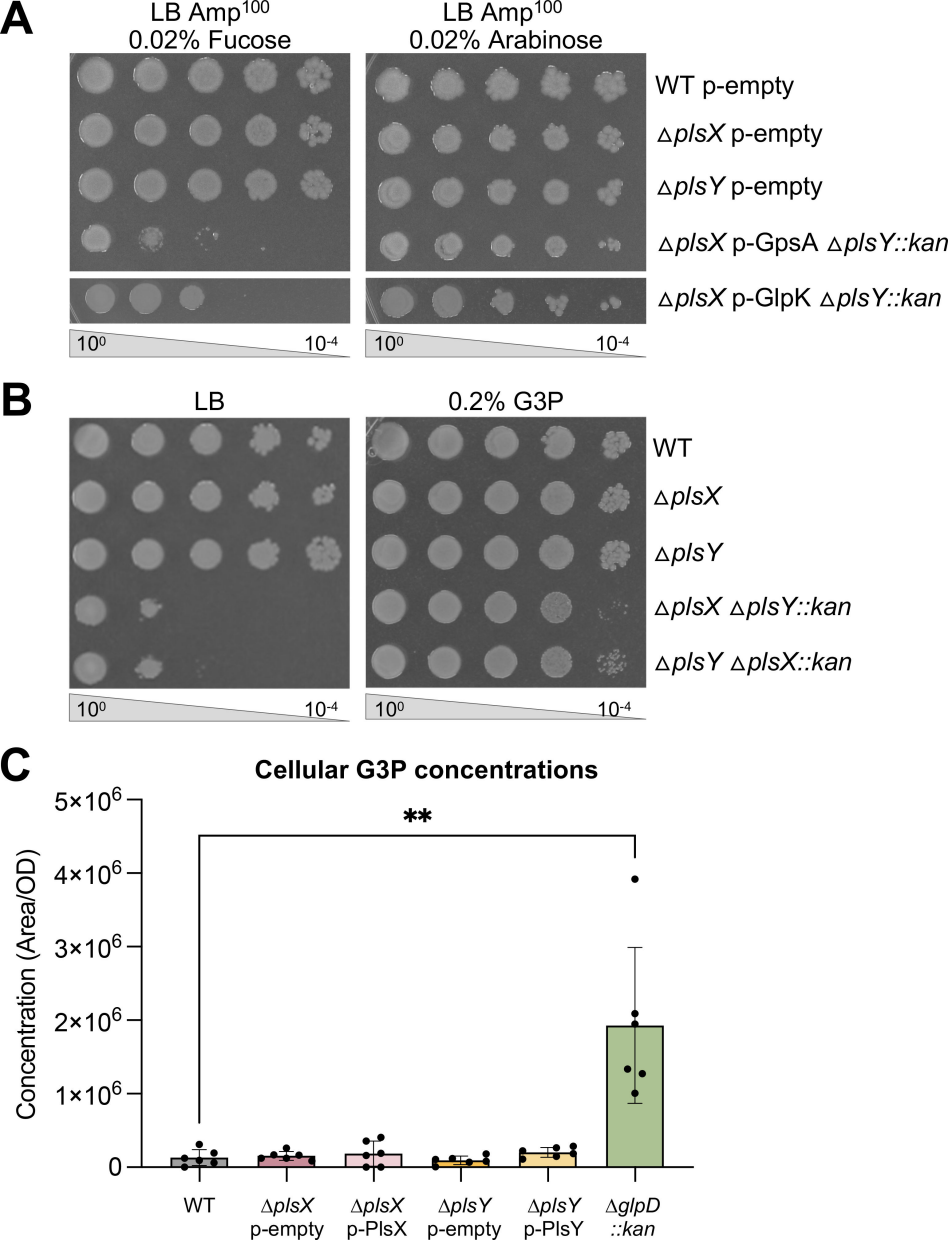
Increasing the level of G3P rescues ∆*plsX* ∆*plsY* synthetic lethality. (**A**) Overexpression of GpsA or GlpK from a plasmid with an arabinose-inducible promoter rescues synthetic lethality of *∆plsX* ∆*plsY*. Serial dilutions of indicated strains were spotted on LB amp in repressing (0.02% fucose) or inducing (0.02% arabinose) conditions and incubated at 37°C. (**B**) Supplementation of G3P rescues synthetic lethality of *∆plsX* ∆*plsY*. Serial dilutions of indicated strains were spotted on LB or LB supplemented with 0.2% G3P and incubated at 37°C. Data in panels A and B are representative of three biological replicates. (**C**) G3P concentration in ∆*plsX* (p-PlsX) and ∆*plsY* (p-PlsY) does not significantly change. As expected, G3P concentration increases in a *glpD::kan* mutant. Error bars in panel C represent SD from six technical replicates, and data are representative of two biological replicates. **, *P* ≤  0.01

To test if increasing G3P levels suppressed ∆*plsXY*, we overexpressed WT alleles of GlpK or GpsA (Fig. 1A inset) to artificially increase G3P pools. Overexpression of GlpK or GpsA rescued growth of ∆*plsXY* by efficiency of plating assays (Fig. 3A). Noga et al. showed that G3P concentration correlated with increased GPL flux in *E. coli*, but supplementing glycerol to media did not correlate with GPL flux, even though total G3P concentration increased (20). We hypothesized that suppressor mutations worked to increase GPL flux, and therefore, supplementation of glycerol would not rescue ∆*plsXY*. Supporting this conclusion, exogenous supplementation with 0.2% G3P suppressed synthetic lethality (Fig. 3B), while supplementation with 0.2% glycerol did not (Fig. S2A). Together, these data indicated that ∆*plsXY* requires supplementation of G3P to survive. We hypothesized that either ∆*plsX* or ∆*plsY* may have reduced levels of G3P, contributing to the synthetic lethal phenotype. Thus, we analyzed total G3P pools via liquid chromatography-mass spectroscopy (LC-MS). To our surprise, single mutants, as well as overexpression of PlsX or PlsY, did not have significant changes in the internal G3P pools (Fig. 3C). As a positive control for the dynamic range of our G3P quantification, a *glpD::kan* mutant, previously shown to increase cellular concentrations of G3P (17), was also assessed and showed an increased concentration of G3P (Fig. 3C).

In *Bacillus subtilis*, a Gram-positive bacterium with only PlsX/Y (YneS/YgiH), it was shown that supplementation with either glycerol or G3P could rescue a temperature-sensitive allele of *plsX* (*yneS-ts*) (12). In this organism, deletion of *plsX* (*yneS*) or *plsY (ygiH*) is lethal, as there is no PlsB to recover LPA synthesis. However, it is worth noting that *B. subtilis* can also generate acyl-PO_4_ from exogenous fatty acids via the FakAB system, providing additional substrate for PlsY. Rescue of the *plsX-ts* mutant with exogenous glycerol or G3P suggests that an increase in PlsY activity was necessary, likely because acyl-PO_4_ concentration was low. It is not surprising that *B. subtilis* and *E. coli* ∆*plsXY* mutants would vary in the ability of glycerol to rescue lethality. We speculate that as a Gram negative, *E. coli* may require tighter control of the conversion of glycerol to G3P to balance the synthesis of OM lipids. Previously, it was shown that a *plsXY* double mutant was viable when PlsB was expressed *in trans* (12). We sought to validate this rescue of Δ*plsXY* synthetic lethality via overexpression of PlsB as it would support that pushing GPL synthesis is essential in this genetic background. A Δ*plsY::kan* allele was introduced via P1 phage transduction into Δ*plsX* while overexpressing PlsB *in trans* and vice versa (∆*plsX::kan* into a ∆*plsY* recipient while overexpressing PlsB *in trans*). Cells were grown under inducing conditions (arabinose) overnight, washed, and switched to repressing (glucose) conditions. PlsB overexpression suppressed the synthetic lethality of the *plsXY* double mutant by efficiency of plating assay (Fig. S2B). Together, rescue by PlsB overexpression and heightened G3P suggested that LPA synthesis needed to be pushed to suppress the lethal phenotype of ∆*plsXY*. These results indicate that PlsXY likely contributes to LPA synthesis, albeit a minor contribution compared to PlsB because individually PlsX and PlsY are not essential. Individual loss of PlsX or PlsY would have the same impact to LPA synthesis as loss of both; therefore, reduced LPA synthesis would have to be only one facet of why *plsX* and *plsY* were synthetically lethal.

### Loss of PlsX results in dysregulation of FASII

**Fig 4:**
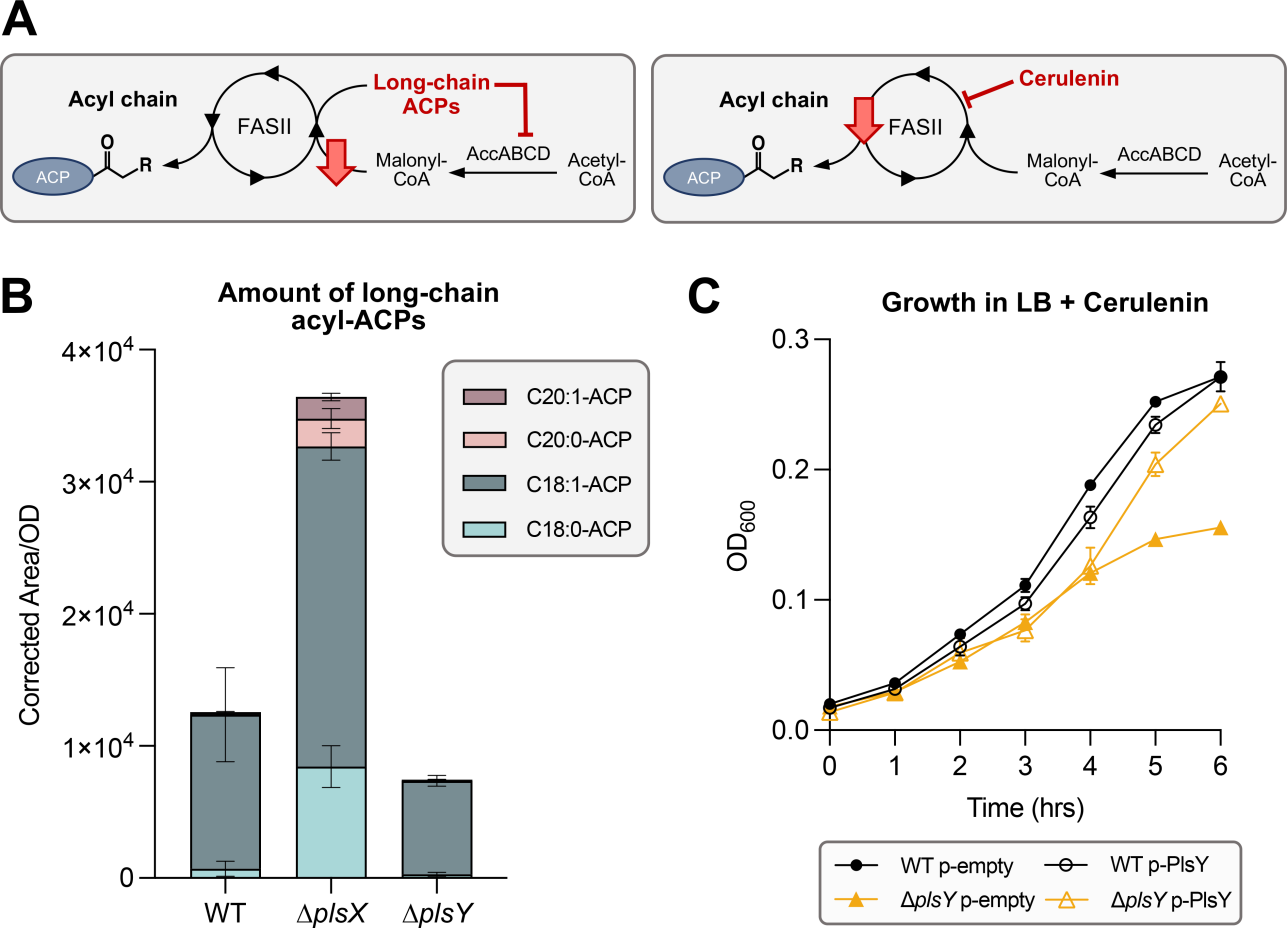
Loss of PlsX causes dysregulation of FASII. (**A**) Build-up of long-chain acyl-ACPs feedback inhibit the AccABCD complex which initiates FASII, ultimately slowing elongation by reducing the level of malonyl-CoA. Cerulenin, an irreversible inhibitor of FabB and FabF, slows FASII. (**B**) Quantification of total long-chain acyl-ACPs in ∆*plsX* shows increased C18:0/1-ACP, as well as the presence of C20:0/1-ACP. The latter is barely detected in both WT and ∆*plsY*. Technical triplicates are shown and are representative of three biological sets. (**C**) Growth curve showing the sensitivity of ∆*plsY* to the presence of 45 µg/mL cerulenin. Overexpression of PlsY *in trans* rescues sensitivity. Strains were grown in LB amp under inducing (100 µM IPTG) conditions. Error bars represent SD from three biological replicates and are not shown when smaller than the symbol.

Next, we tried to further distinguish what additional phenotypes contributed to the ∆*plsXY* synthetic lethality. We hypothesized that ∆*plsXY* may have an imbalance in other GPL precursors, namely acyl-ACPs. Alterations to the quantity and composition of acyl-ACP pools are a known regulatory mechanism for both the total amount of fatty acid synthesis in the cell and for the balance between GPL and LPS synthesis (21). Fatty acid precursors are finely tuned during elongation via FASII as enough shorter chain acyl-ACPs (C12 and C14) are needed to support LPS synthesis, while sufficient fatty acids must continue through elongation to produce longer acyl-ACPs (C16 and C18) required for GPL synthesis. Proper LPS/GPL synthesis is maintained by the balanced activities of LpxC (deacetylase) that commits C14-ACPs to LPS synthesis (22) and FabZ (dehydratase) that continues the elongation of acyl-ACPs for GPL synthesis (23). Additional balance is maintained through competition for fatty acids between GPL synthesis and FASII. If acyl-ACPs are not utilized for GPL synthesis, they can continue to be elongated. Accumulation of long-chain acyl-ACPs (e.g., C20-ACP) indicates that the incorporation of fatty acids into GPLs has slowed, providing a cellular signal that FASII flux is too high or indicating a decreased cellular demand for GPL synthesis. Such long-chain acyl-ACPs participate in feedback inhibition by binding to the FASII initiation complex, AccABCD (ACC), reducing FASII flux (Fig. 4A) (13). ACC generates malonyl-CoA, a metabolite that is solely consumed by FASII, which is converted to malonyl-ACP by FabD to prime for elongation.

To test if ∆*plsX* or ∆*plsY* impacted FASII, we quantified the pool of acyl-ACPs. While malonyl-ACP concentrations are highly sensitive to FASII flux (20), there are three possible alterations to the elongated acyl-ACP pools that would support distinct hypotheses for the role of one of our proteins. The first possible result was that total acyl-ACPs could be decreased, suggesting that either PlsX or PlsY may help to stimulate a step of FASII initiation. A second possibility was that there could be an accumulation of medium-chain (C12 and C14) acyl-ACPs, which may increase LPS flux and reduce the quantity of acyl-ACPs being elongated for GPL flux. This result would suggest that PlsX or PlsY was critical for regulating the balance between LPS and GPLs, possibly through impacting LpxC or FabZ activity. The third possibility was that longer chain (C18 and C20) acyl-ACPs could accumulate, which would concomitantly result in feedback inhibition of FASII initiation. This result would suggest that PlsX or PlsY had a role in preventing long-chain acyl-ACP accumulation.

Loss of PlsY had no changes to the overall long-chain acyl-ACP pools—in terms of the presence of new acyl-ACP species (Fig. 4B). In contrast, loss of PlsX resulted in a profile consistent with the third hypothesis—increased C18 acyl-ACPs and appearance of C20 long-chain acyl-ACPs (Fig. 4B). Overexpression of PlsX *in trans* in ∆*plsX* recovered the accumulation of long-chain acyl-ACPs (Fig. S3). This complementation in ∆*plsX* also supports the lack of polar effects on FASII genes downstream of *plsX*. Overexpression of PlsY in ∆*plsY* did not have any impact on the quantity of long-chain acyl-ACPs in the cell (Fig. S3). Acyl-ACPs of 16-, 18-, and 20-carbon length have been shown to inhibit ACC (13). This result suggested that PlsX has a regulatory role in preventing the accumulation of long-chain acyl-ACPs in a manner that is independent of the production of LPA because this impact is not seen in Δ*plsY* which would have the same, albeit minor, reduction of LPA production as ∆*plsX*. These results are consistent with PlsX and PlsY each having independent cellular roles in addition to their combined role in LPA synthesis.

We sought to determine if the accumulation of long-chain fatty acids in ∆*plsX* was enough to substantially repress FASII initiation and therefore cause synthetic lethality of a ∆*plsXY* double mutant. To test this, we utilized the antibiotic cerulenin which slows FASII by irreversibly inhibiting FabB/F/H, three FASII enzymes crucial for elongation of fatty acids (Fig. 4A) (24, 25). This inhibition of FASII would mimic the possible ACC inhibition from long-chain acyl-ACP accumulation in *plsX* mutants. The *plsY* mutant was hypersensitive to inhibition by cerulenin, indicating that other mechanisms of slowing FASII, like accumulation of long-chain fatty acids in ∆*plsX*, are toxic when combined with *plsY* deletion (Fig. 4C). Cerulenin hypersensitivity of ∆*plsY* was also complemented by expressing PlsY *in trans* (Fig. 4C). Together, these results suggested that PlsX has an unprecedented role in fine-tuning FASII to prevent the accumulation of long-chain acyl-ACPs.

### PlsX actively impacts FASII via FadD

**Fig 5:**
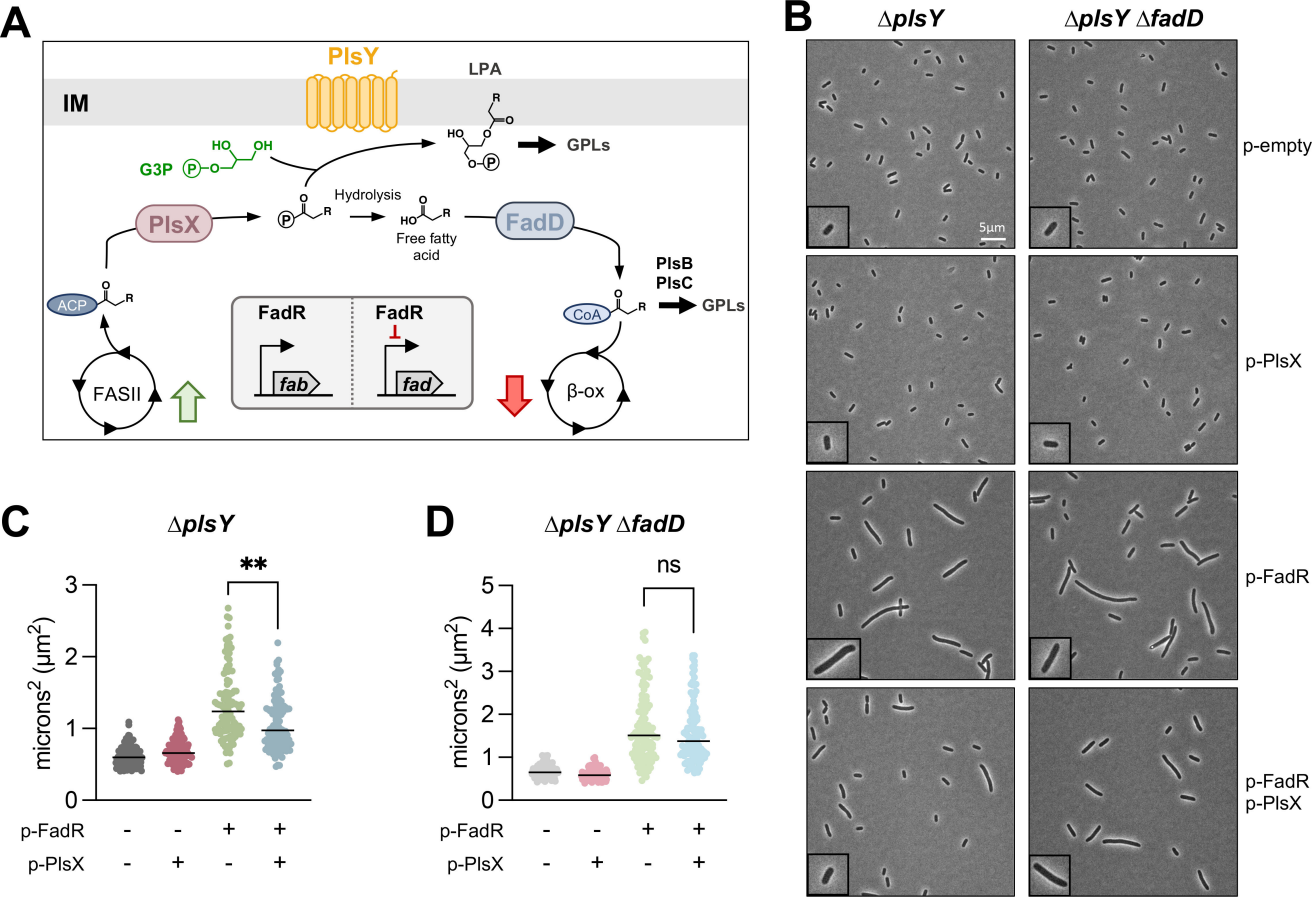
Overexpression of PlsX reduces increased cell size when FASII flux is upregulated. (**A**) In *E. coli*, FASII and β-oxidation (β-ox) are highly regulated pathways for synthesis and degradation of acyl-ACPs or acyl-CoAs, respectively. One such enzyme for regulation is FadR, which activates transcription of FASII enzymes while repressing transcription of β-ox enzymes. PlsX pulls acyl-ACP from FASII, generating an acyl-PO_4_ which can be used by PlsY for GPL synthesis or spontaneously degraded to a free fatty acid. FadD converts free fatty acids to acyl-CoAs for degradation through β-ox. If the acyl-CoAs are of appropriate length for GPL synthesis, PlsB/C can utilize them. (**B**) Phase contrast microscopy of ∆*plsY* and ∆*plsY* ∆*fadD* with overexpression of FadR and PlsX at 1,000× magnification. Scale bar is 5 µm. A single cell from the field of view is highlighted with a ×2 magnified inset. (**C**) Morphological changes associated with overexpression of FadR in ∆*plsY* are partially rescued by simultaneous overexpression of PlsX as shown via a decrease in overall cell area. (**D**) Decrease in area by PlsX when FadR is overexpressed is abolished when FadD is deleted, suggesting this response is FadD-mediated. Data in panels C and D represent mean values with the SD from >5 fields of view and >100 cells per strain. Data are representative of biological triplicates. For panels B–D, OD_600_ for cultures was within 10% variance. Significance was calculated using Brown-Forsythe and Welch ANOVA tests. **, *P* ≤  0.01; ns, not significant.

How might PlsX mediate alterations in the fatty acid pool? If PlsX is consuming long-chain acyl-ACPs (16C and 18C) in parallel with PlsB, then it acts to effectively decrease the average chain length of acyl-ACPs undergoing elongation due to a shifting in the balance between long-chain acyl-ACP elongation (FASII) and incorporation into GPLs (via PlsY). This modulation would occur as FASII acts to balance the appropriate length of acyl-ACP with cellular demand—C12/14 for LPS synthesis and C16/18 for GPL synthesis—while also working to prevent elongation to C20. PlsX might also influence the fatty acid pool by the production of free fatty acids (via hydrolysis). Typically, PlsX converts an acyl-ACP into an acyl-PO_4_, a substrate used solely by PlsY. However, these acyl-PO_4_(s) are highly labile and can hydrolyze to a free fatty acid if not used quickly (26). The free fatty acid can then be converted into an acyl-CoA by FadD, a fatty acid-CoA ligase, and be degraded through β-oxidation (Fig. 5A) (27). In this way, PlsX activity may help to control the level of acyl-ACPs within the cell.

To test if PlsX was capable of redirecting excess acyl-ACPs into β-oxidation, we sought a genetic background with excessive production of acyl-ACPs. When FadR (DNA-binding dual regulator) is present and in an active conformation, FASII genes are activated and β-oxidation genes are downregulated to balance the synthesis and degradation of acyl-chains. However, upon binding an acyl-CoA, FadR-DNA interactions are disrupted, resulting in decreased expression of FASII genes, a reduction in acyl-ACP pools, and derepression of β-oxidation genes (28). Overexpression of FadR increases fatty acids that are of appropriate length for GPL synthesis, leading to increased lipid membrane content that correlated with increased cellular area (29). We hypothesized that overexpression of PlsX would rescue this phenotype by pulling excess acyl-ACPs from FASII and pushing flux toward FadD, generating acyl-CoAs (Fig. 5A). While FadR would act to repress *fad*, there are still basal levels of *fad* transcripts due to product feedback inhibition on FadR. To ensure overexpression of PlsX does not push acyl-PO_4_ toward GPL synthesis, we performed these experiments in a *plsY* mutant.

Overexpression of PlsX alone in ∆*plsY* did not alter cell size, suggesting that acyl-CoAs generated through FadD are either not being utilized by PlsB/C for GPL synthesis or are produced in too little quanitity to detect global changes (Fig. 5B). With FadR overexpression, cells behave as expected, showing an overall increase in cell area and length. In ∆*plsY* overexpressing both FadR and PlsX, there is visible partial rescue in cell size confirmed by cell measurements (Fig. 5B and C; Fig. S4A through C). We contribute incomplete rescue to the complex regulation occurring within the cell regarding FadR. Due to feedback inhibition of acyl-CoAs on FadR, β-oxidation proteins would rise and decrease acyl-CoA pools, thereby relieving FadR inhibition—forming a bottleneck at FadD for acyl-CoA degradation.

To test if rescue of ∆*plsY* overexpressing both FadR and PlsX was FadD mediated, we repeated microscopy in ∆*plsY* ∆*fadD* and saw that PlsX overexpression failed to rescue FadR overexpression defects (Fig. 5B and D). These findings were confirmed by measuring changes in cell morphology (Fig. 5C and D; Fig. S4A through C) and suggest that PlsX may work to fine-tune FASII in a FadD-dependent manner—whether that be by increasing β-oxidation or through feedback regulation of FadR. Altogether, these data suggested that PlsX helps to prevent the accumulation of fatty acids.

### Suppressors that rescue growth of ∆*plsXY* do not rescue phenotypes associated with single *plsX* and *plsY* mutants

With a possible secondary role for PlsX identified, we were curious if suppressor mutations that restore growth to ∆*plsXY* would rescue phenotypes observed when only *plsX* or *plsY* was absent. Deletion of *glpD* increased G3P levels (Fig. 3D), and given that increased G3P correlates with increased GPL flux (20), we suspected that loss of *glpD* would rescue ∆*plsXY*. We first aimed to confirm that ∆*glpD* rescues synthetic lethality of ∆*plsXY* through co-transduction linkage (∆*plsY* ∆*ygjH::kan*). We found that co-transduction of the Δ*plsY* allele linked to Δ*ygjH::kan* was possible into a ∆*plsX ∆glpD* strain, showing that ∆*glpD* does rescue lethality (Table S1). Next, we questioned if *glpD* deletion would rescue the secondary impacts of losing PlsX and PlsY individually and introduced a *glpD* deletion into both ∆*plsX* and ∆*plsY*.

**Fig 6:**
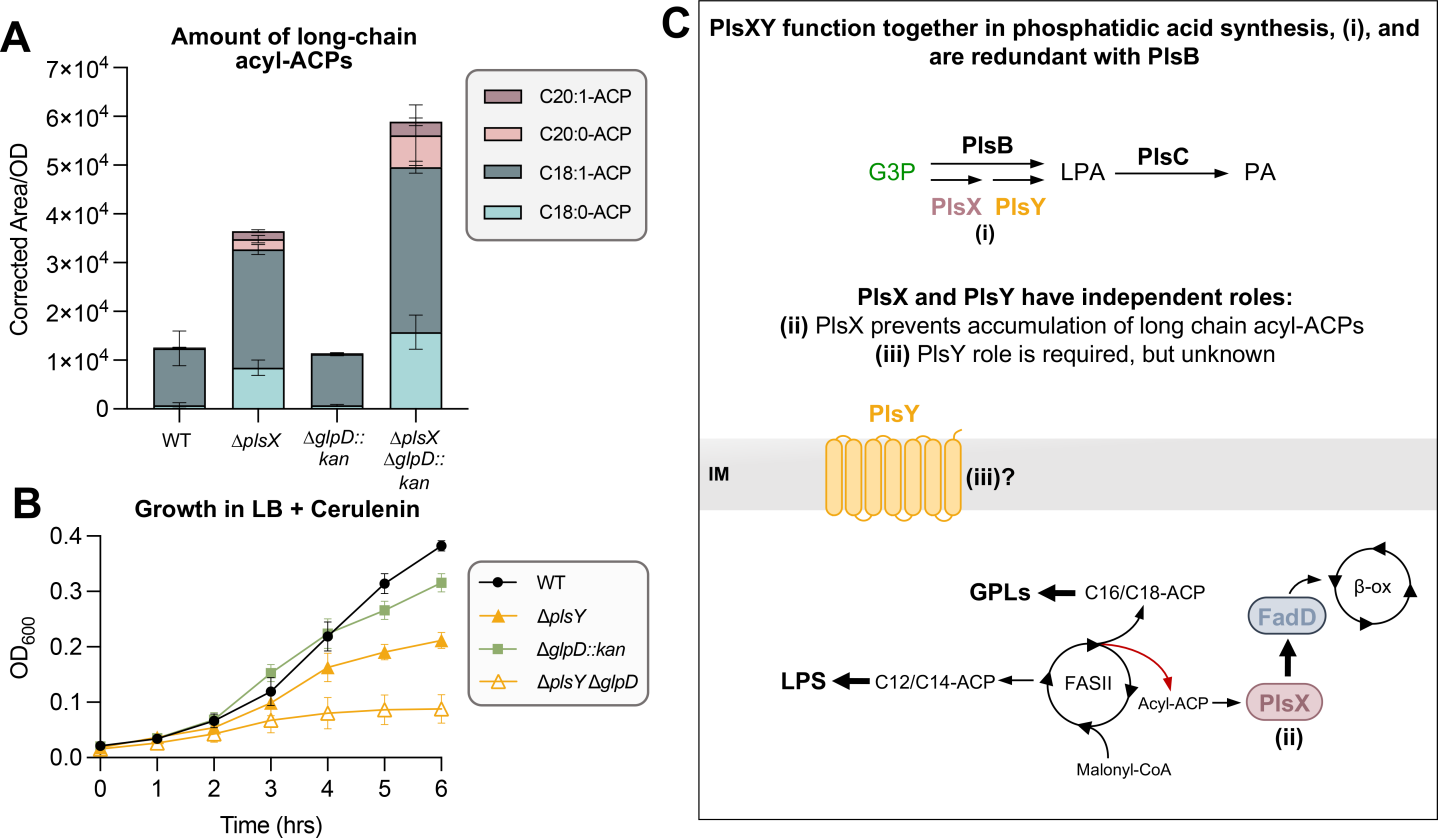
Three biological roles contribute to synthetic lethality of ∆*plsXY*. (**A**) Quantification of total long-chain acyl-ACPs in ∆*plsX* and ∆*plsX* ∆*glpD::kan* shows increased C18:0/1-ACP, as well as the presence of C20:0/1-ACP. The latter is barely detected in both WT and ∆*glpD::kan*. Error bars represent SD from three technical replicates. (**B**) Growth curve showing the sensitivity of ∆*plsY* to the presence of 45 µg/mL cerulenin. Deletion of ∆*glpD::kan* grows similarly to WT, while ∆*plsY* ∆*glpD* does not rescue growth in the presence of cerulenin. Error bars represent SD from three biological replicates and are not shown when smaller than the symbol. (**C**) PlsXY has redundant roles with PlsB for synthesis of PA (i). Data presented here suggests that PlsX prevents accumulation of long-chain acyl-ACPs in a FadD-dependent manner (ii), while PlsY must play an additional, but unknown tertiary role (iii).

Total acyl-ACPs levels were quantified in ∆*glpD::kan* and ∆*plsX* ∆*glpD::kan* to determine if increasing G3P levels would reduce long-chain acyl-ACP build-up when PlsX was absent. As shown earlier in Fig. 4B, ∆*plsX* has an increase in C18:0/1-ACPs, as well as the appearance of C20:0/1-ACPs, whereas the deletion of *glpD* alone had no effect (Fig. 6A). Deletion of *glpD* in ∆*plsX* did not restore WT levels of long-chain acyl-ACPs (Fig. 6A). To test whether deletion of *glpD* could impact a *plsY*-specific phenotype, we evaluated the *plsY* mutant’s sensitivity to cerulenin. Cerulenin slightly decreased the growth of *∆glpD*; however, deletion of *glpD* did not rescue sensitivity of ∆*plsY* (Fig. 6B). Together, these data supported that ∆*plsXY* suppressors do not restore the additional roles of PlsX and PlsY outside of the canonical pathway for LPA synthesis.

## DISCUSSION

By the mid-90s, the majority of the enzymatic machinery required for FASII and PA synthesis in Gram-negative bacteria had been identified, including PlsB and PlsC. However, the complex mechanisms governing these processes, as well as some of the key enzymatic players, remained unknown. Specifically, PlsX was a known acyltransferase, though not well-studied (30), and PlsY remained undiscovered. Consequently, dogma of that time centered PlsB/C as the traditional pathway for PA synthesis. It was not until 10 years later that Lu et al. (5) discovered the novel acyltransferase PlsY in Gram-positive bacteria. This discovery “Rock”-ed the field by demonstrating that the PlsX/Y pathway was the most wide-spread system for initiation of PA synthesis in bacteria, not PlsB. Interestingly, it became clear that various species of proteobacteria evolved to maintain both PlsB and PlsX/Y (8). This conservation piqued the question of why some Gram-negative species require a seemingly redundant system?

In Gram-negative bacteria, PlsB is more deeply studied than PlsX and PlsY (31) since single *plsX* and *plsY* deletions are viable and *plsB* is essential. The synthetic lethality of the *plsXY* mutant has remained understudied. Because disabling either enzyme eliminates the PlsX/Y route to PA synthesis, synthetic lethality suggests that PlsX and PlsY have multiple roles in the Gram-negative cell outside of the canonical PA synthesis pathway (Fig. 6C). Based on our data, we propose three roles of PlsX/Y that together contribute to the synthetic lethality. Gram-positive and Gram-negative PlsX/Y contain high conservation and homology, suggesting maintenance of the enzymatically proven activity (5, 32). Furthermore, our suppressors (discussed below) of *plsXY* synthetic lethality would all push PlsB activity, suggesting that a contribution to LPA synthesis is one facet of the synthetic effect. Thus, for role (i), we agree with existing literature that PlsX and PlsY together participate in PA production, although in minor quantities (Fig. 6C). For role (ii), our data suggest that PlsX in Gram-negative bacteria prevents accumulation of long-chain acyl-ACPs and actively connects FASII and β-oxidation in a FadD-dependent manner (Fig. 6C, discussed further below). Finally, because single ∆*plsX* mutants are viable and would be missing role (i and ii), we conclude that PlsY must have a third role in the cell that has yet to be discovered and contributes to the synthetic lethality of *plsXY* double mutants [Fig. 6C, role (iii)]. This potential additional role of PlsY is actively under investigation in our laboratory. Since Δ*plsY* would have partial disruption of PA synthesis [role (i)] along with loss of role (iii), we conclude that the loss of all three roles has to contribute to synthetic lethality (Fig. 6C). Altogether, our findings support that PlsX and PlsY contribute to three roles in the cell, and it is the loss of all three roles that is synthetically lethal.

We validated that overexpression of PlsB (Fig. S2B) rescued synthetic lethality of Δ*plsXY*, suggesting that a reduction of PA synthesis [role (i)] contributed to lethality. This conclusion was also supported by rescue with GlpK or GpsA overexpression (artificially increasing G3P concentration; Fig. 3A) and exogenous supplementation of G3P (Fig. 3B). Heath and Rock (33) worked with isolated mutants that depend on G3P for growth (34) and characterized the *plsB26* allele which resulted in a variant with elevated *K*_m_ for G3P. However, this allele alone did not cause cells to become dependent on G3P. A second frameshift mutation in *plsX* (*plsX50*) (35) combined with *plsB26* resulted in a G3P-dependent strain (36). These findings suggested that if PlsB activity is compromised, PlsX activity is able to contribute to PA synthesis [role (i)]. Our work similarly found that increasing G3P and altering flux toward PA synthesis rescued the growth of the *plsXY* double mutant.

Outside of role (i), we propose that PlsX functions to connect FASII and β-oxidation [role (ii)]. This role would be specific to Gram-negatives as many Gram-positive bacteria do not contain homologous genes for β-oxidation (37) and others have incomplete or undiscovered pathways (8). While previous work suggested a role for PlsX in acyl-ACP regulation (12), we further propose that PlsX works in a FadD-dependent manner to prevent the accumulation of longer chain acyl-ACPs (e.g., C20:0-ACP). PlsX pulls acyl-ACP from FASII. If the cell needs production of GPLs, then these intermediates would be quickly consumed by PlsY. However, if PlsY activity is low or inhibited then the acyl-PO_4_ would spontaneously dephosphorylate into a free fatty acid. Free fatty acids can then be converted to acyl-CoA’s by FadD for reutilization or degradation. Supporting this theory, overexpression of the thioesterase TesA has been reported to rescue a ∆*plsXY* mutant (12). TesA is normally a periplasmic thioesterase, but overexpression was found to result in trapping of the enzyme in the cytoplasm, allowing for cleavage of acyl-thiols. In this way, cytoplasmic TesA cleaves acyl-ACPs similar to the hypothesized turnover of acyl-PO_4_ via PlsX. TesA overexpression would therefore rescue a ∆*plsX* ∆*plsY* mutant as it would restore loss of role (ii).

FadD-generated acyl-CoA molecules have three possible directions in Gram-negative cells. (i) PlsB and PlsC may utilize an acyl-CoA of appropriate lengths for PA synthesis. (ii) The acyl-CoA may be degraded through β-oxidation. (iii) FadR may bind to the longer chain acyl-CoA, causing changes to regulation of both FASII (de-activation) and β-oxidation (de-repression). Overall, these data suggest that *E. coli* produces more acyl-ACPs than are required and that PlsX is needed to prevent the accumulation of long-chain acyl-ACP by maintaining a proper balance between fatty acid elongation and incorporation into GPLs.

Role (iii) of PlsY has yet to be elucidated. Recently, PlsY was demonstrated to interact with YejM (38), a regulator of LPS synthesis. This is a new and rapidly expanding field of research. LpxC is proteolytically regulated by the adapter protein LapB and the protease FtsH (21). LapB is occluded through interaction with an IM sensor, YejM, and released when LPS accumulates in the outer leaflet of the IM, allowing for degradation of LpxC (21). In addition to serving as an adaptor for FtsH cleavage, LapB was also recently shown to bind and inhibit LpxC activity (39), indicating the many nuances to this tight regulation. We are actively working to elucidate the role that PlsY may play in modulating the activity of YejM, or vice versa. Altogether, our findings suggest that maintenance of the PlsX/Y pathway in Gram-negative organisms provides a fitness advantage by allowing the cell to fine-tune the level of major lipids. This control is critical for overall cell envelope homeostasis and for maintaining OM asymmetry.

The choice to exploit PlsB or PlsX/Y as the major LPA synthesis pathway also conveniently correlates with differences in how Gram-negatives and Gram-positives utilize exogenous fatty acids from the environment. In Gram-positives, which possess only PlsX/Y, exogenous fatty acids are converted to acyl-PO_4_ by the FakA/B system (27), which can be used by PlsY. Thus, the combination of PlsY and FakA/B allows Gram positives to grow on exogenous fatty acids. The robust outer membrane of Gram-negative species requires the import of exogenous fatty acids through FadL/D, which instead converts fatty acids into acyl-CoAs. PlsB can utilize acyl-CoAs, whereas PlsX/Y cannot. Therefore, the combination of PlsB and FadL/D achieve the same goal of utilization of exogenous fatty acids for Gram-negatives. By utilizing PlsB to drive the majority of LPA synthesis from both *de novo* synthesized (acyl-ACPs) and exogenous (acyl-CoA) fatty acids, perhaps Gram-negative organisms like *E. coli* were then free to repurpose PlsX/Y in ways to balance lipid synthesis and promote outer membrane stability.

## MATERIALS AND METHODS

### Bacterial growth conditions

Bacteria were grown in LB or on LB agar at 37°C with appropriate supplementation—ampicillin (amp; 100 µg/mL), kanamycin (kan; 30 µg/mL), chloramphenicol (cam; 30 µg/mL), cerulenin (45 µg/mL), L-arabinose (wt/vol), D-glucose (wt/vol), D-fucose (wt/vol), glycerol (vol/vol), or G3P (wt/vol) as indicated in figure legends. For growth curves, bacteria were grown in 20 mm test tubes with 5 mL of media, unless otherwise stated, and OD_600_ was measured with 150 µL of culture in a 96-well plate on the BioTek Epoch 2 plate reader.

### Strain and plasmid construction in *E. coli*

All strains, plasmids, and oligonucleotides used in this study are listed in the Data Set S1. Strains were generated using P1 phage transduction from Keio collection strains as previously described (40). Kanamycin cassettes were removed using flippase/flippase recognition target site-specific recombination activity from the pCP20 plasmid as previously described (41). *plsX*, *glpK*, *gpsA*, and *plsB* were cloned into pCV1 or pCV3 plasmids utilizing BspQI cloning as previously described (42). Briefly, W3110 genomic DNA was isolated with the Easy-DNA (Invitrogen) kit and the genes of interest amplified by PCR using their respective primers. PCR products were digested and ligated with plasmid using the Fast-Link DNA Ligation Kit (Avantor) and transformed into DH5α. Construction of pWSKI was previously reported (43). Briefly, pBAD18::*fadR* (EcoRI and KpnI) was generated via restriction enzyme cloning by digestion, ligation, and transformation into DH5α. Genscript services were used for subcloning of PlsX (NotI and BamHI) and PlsY (NotI and EcoRI) into pWSKI. Plasmids were verified using whole-plasmid sequencing (Plasmidsaurus).

### Depletion growth curve for demonstrating ∆*plsX ∆plsY* synthetic lethality

One mL of overnight bacterial cultures in LB amp 0.02% glucose was washed twice and resuspended in 1 mL of fresh LB. Bacteria were inoculated to an OD_600_ 0.001 in 200 µL of appropriate media in a 96-well plate and incubated for 3 hours at 37°C with shaking. Cultures were back-diluted again to an OD_600_ 0.001 in fresh media and incubated for an additional 2 hours. Cultures were back-diluted once more to OD_600_ 0.01 and incubated at 37°C.

### Efficiency of plating assays

Overnight bacterial cultures were standardized by OD_600_ and serially diluted 1:10 in LB media in a 96-well plate. Dilutions were spotted onto the indicated LB antibiotic plates with a Replica Plater for 96 well plate (Sigma-Aldrich) and incubated for 16 hours at 37°C.

### RNA-seq analysis

Overnight bacterial cultures were back-diluted in 5 mL of LB to an OD_600_ 0.05 and incubated to an OD_600_ ~0.8. One mL of culture was combined with 2 mL of RNAprotect (QIAGEN), vortexed for 15 seconds, and incubated at room temperature for 5 minutes. The mixture was centrifuged at 5,000 × *g* for 10 minutes and the supernatant discarded. Samples were prepped in biological triplicates. Library preparation, rRNA depletion, and RNA sequencing were carried out by SeqCenter, LLC using Illumina RNA Sequencing with ~12 M RNA reads/sample. RNA-seq analysis was performed using QIAGEN CLC Genomics Workbench.

### Selection of suppressors that restore growth to *plsXY* double mutants

Overnight cultures of either ∆*plsX* or ∆*plsY* were used as the recipient strain for generalized P1 phage transduction with ∆*plsX::kan* or ∆*plsY::kan* generated from the Keio collection. Strains were plated on LB kan supplemented with 5 mM NaCitrate and incubated until colonies formed at 37°C. After isolation, suppressors were cultured in 5 mL of LB, and 1 mL of pelleted overnight culture was sent to SeqCenter, LLC for DNA extraction and sequencing using Illumina Whole-Genome Sequencing with 200 Mbp/sample of data resulting in ~1.5 M reads. Sequencing analysis was preformed using QIAGEN CLC Genomics Workbench and *E. coli* W3110 DNA sequence.

### Selection of *plsXY* double mutants in the presence of G3P

Overnight cultures of either ∆*plsX* or ∆*plsY* were used as the recipient strain for generalized P1 phage transduction with ∆*plsX::kan* or ∆*plsY::kan* generated from the Keio collection. Strains were plated on LB kan supplemented with 5 mM NaCitrate and 0.2% G3P and incubated until colonies formed at 37°C. After isolation, suppressors were cultured for 6 hours in 5 mL of LB supplemented with 0.2% G3P, and 1 mL of culture was sent for WGS as described above. For efficiency of plating assays, 0.2% G3P was supplemented in the overnight cultures, and cells were washed twice before plating.

### Co-transduction for synthetic lethality and rescue of a *plsXY* double mutant

Overnight cultures of either W3110, ∆*plsX*, ∆*plsY*, or ∆*plsX* ∆*glpD* were used as the recipient strain for generalized P1 phage transduction with ∆*plsX* ∆*trhO::kan* or ∆*plsY* ∆*ygjH::kan* generated using the Keio collection. Strains were plated on LB kan supplemented with 5 mM NaCitrate and incubated until colonies formed at 37°C. Approximately 30 colonies were patched to LB kan and incubated overnight at 37°C. Patches were PCR screened for gene deletion and gene duplication.

### LC/MS quantification of ACP intermediates and analysis of G3P

Samples were collected and analyzed using a previously reported method (20).

### Phase-contrast microscopy

Overnight cultures were back-diluted to an OD_600_ ~0.05. Strains were incubated for 5 hours (OD_600_ was within 10% variance) in the presence of amp cam and 0.2% arabinose, and 1 µL was spotted onto a coverslip and an LB agar pad placed on top. Cells were imaged at 1,000× magnification on a Nikon Elipse T*i*2 equipped with Orca-Fusion Digital Camera C14440 in phase-contrast. Calibration was performed with a 1 mm ruler and 0.01 mm divisions of an Azzota Corp. micrometer slide. Measurements were performed on >5 fields of view and >100 cells per strain in biological triplicate as previously described (44).

## SUPPLEMENTAL MATERIAL

Data Set S1 mbio.02969-24-s0001.xlsx Description of strains, plasmids, and RNA-seq data.

Supplemental Material mbio.02969-24-s0002.pdf Figures S1 to S4; Table S1.

ASM does not own the copyrights to Supplemental Material that may be linked to, or accessed through, an article. The authors have granted ASM a non-exclusive, world-wide license to publish the Supplemental Material files. Please contact the corresponding author directly for reuse.
