# Supplementary material for: PlsX and PlsY: Additional roles beyond glycerophospholipid synthesis in Gram-negative bacteria
Source: mBio. 2024 Oct 30;15(12):e02969-24. doi: 10.1128/mbio.02969-24 (PMC11633183; doi:10.1128/mbio.02969-24)
Supplement: Supplemental Material — Figures S1 to S4; Table S1. [file mbio.02969-24-s0002.pdf]

Supplemental material supporting "PlsX and PlsY: Additional roles beyond glycerophospholipid synthesis in Gram-negative bacteria" by Rex, et al.

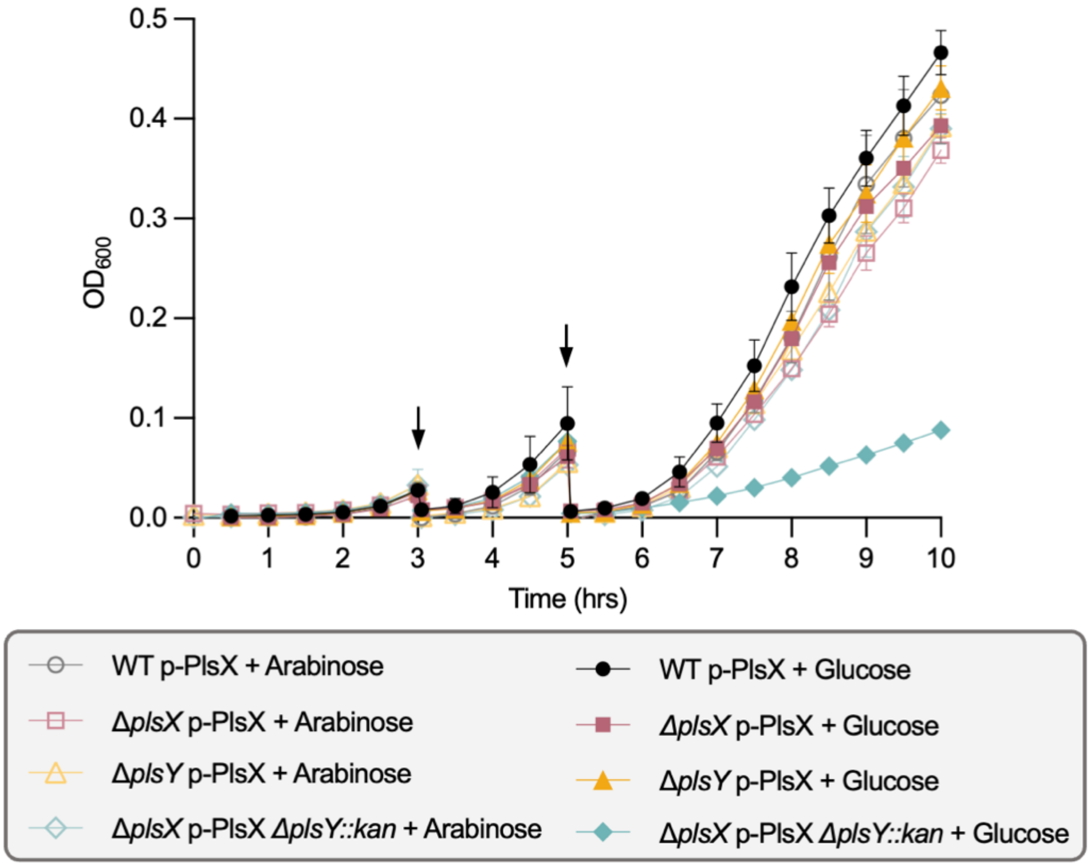

FIG S1 Depletion growth curve showing synthetic growth defect of Δ*plsXY*. Strains were grown in LB amp under inducing (0.05% arabinose) or repressing (0.05% glucose) conditions. Two back-dilutions (denoted by arrows) were performed at 3 and 5 hours to sufficiently deplete plasmid-produced PlsX. Error bars represent SD from three biological replicates and are not shown when smaller than the symbol.

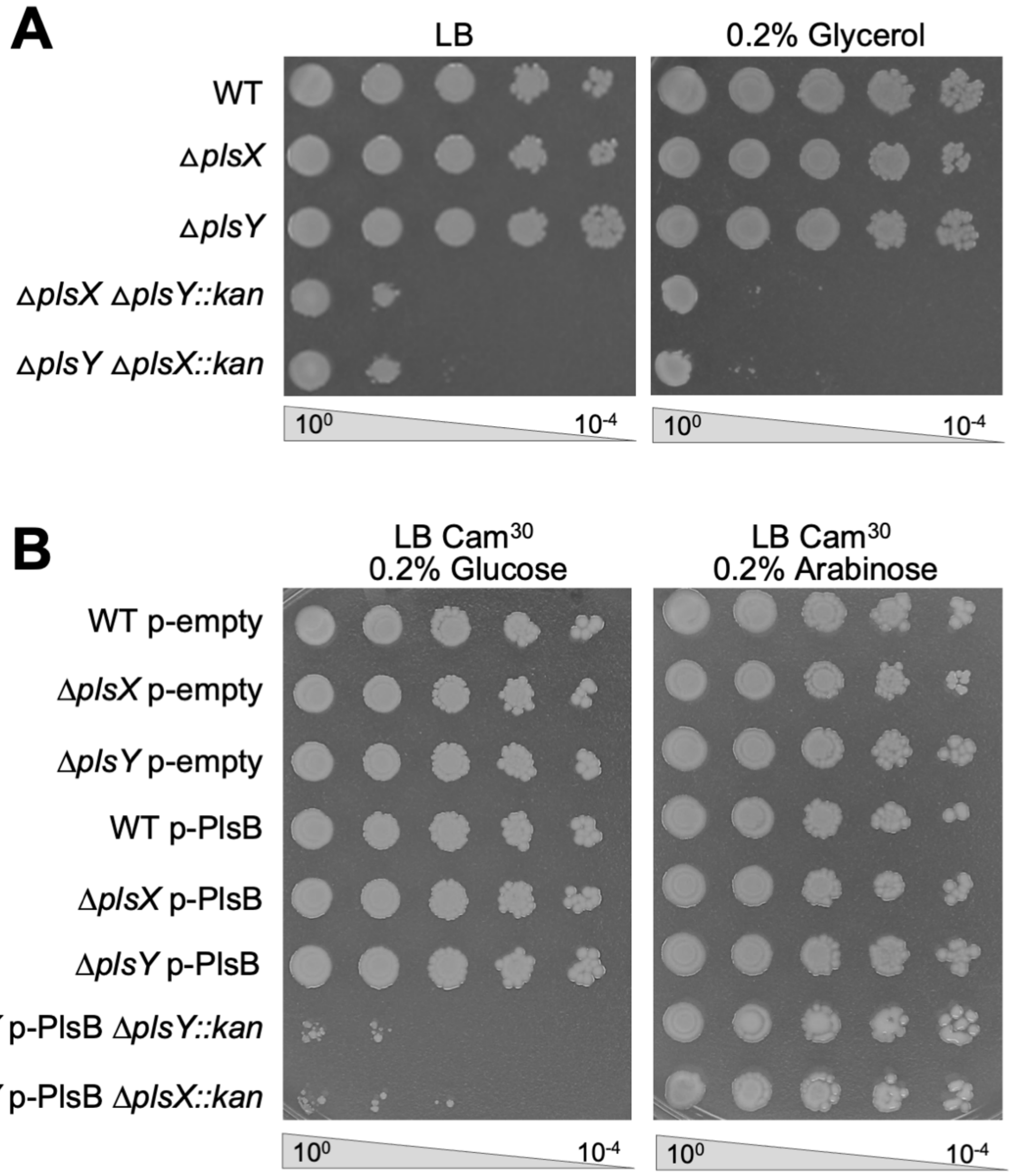

FIG S2 Glycerol supplementation and PlsB overexpression effects on Δ*plsXY* synthetic lethality. (A) Supplementation of glycerol does not rescue synthetic lethality of Δ*plsX* Δ*plsY*. Serial dilutions of indicated strains were spotted on LB or LB supplemented with 0.2% glycerol and incubated at 37°C. (B) Overexpression of PlsB from a plasmid with an arabinose-inducible promoter rescues synthetic lethality of Δ*plsXY*. Serial dilutions of indicated strains were spotted on LB cam under repressing (0.2% glucose) or inducing (0.2% arabinose) conditions and incubated at 37°C. Data in panels A and B are representative of three biological replicates

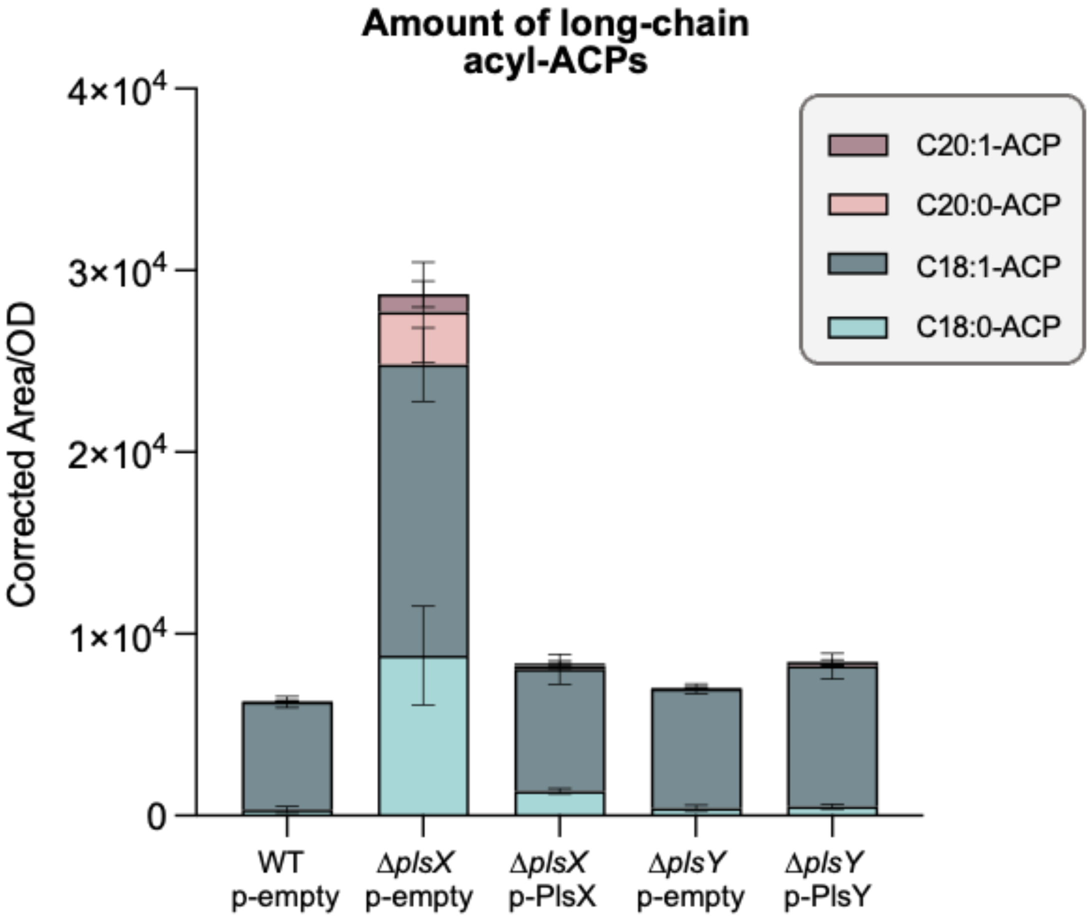

FIG S3 Long chain acyl-ACP pools in WT, ∆*plsX*, and ∆*plsY* with complementation. Levels of long chain acyl-ACPs are shown for acyl chains containing C18:0-ACP, C18:1-ACP, C20:0-ACP, and C20:1-ACP. Error bars represent SD from three technical replicates, data is representative of two biological replicates.

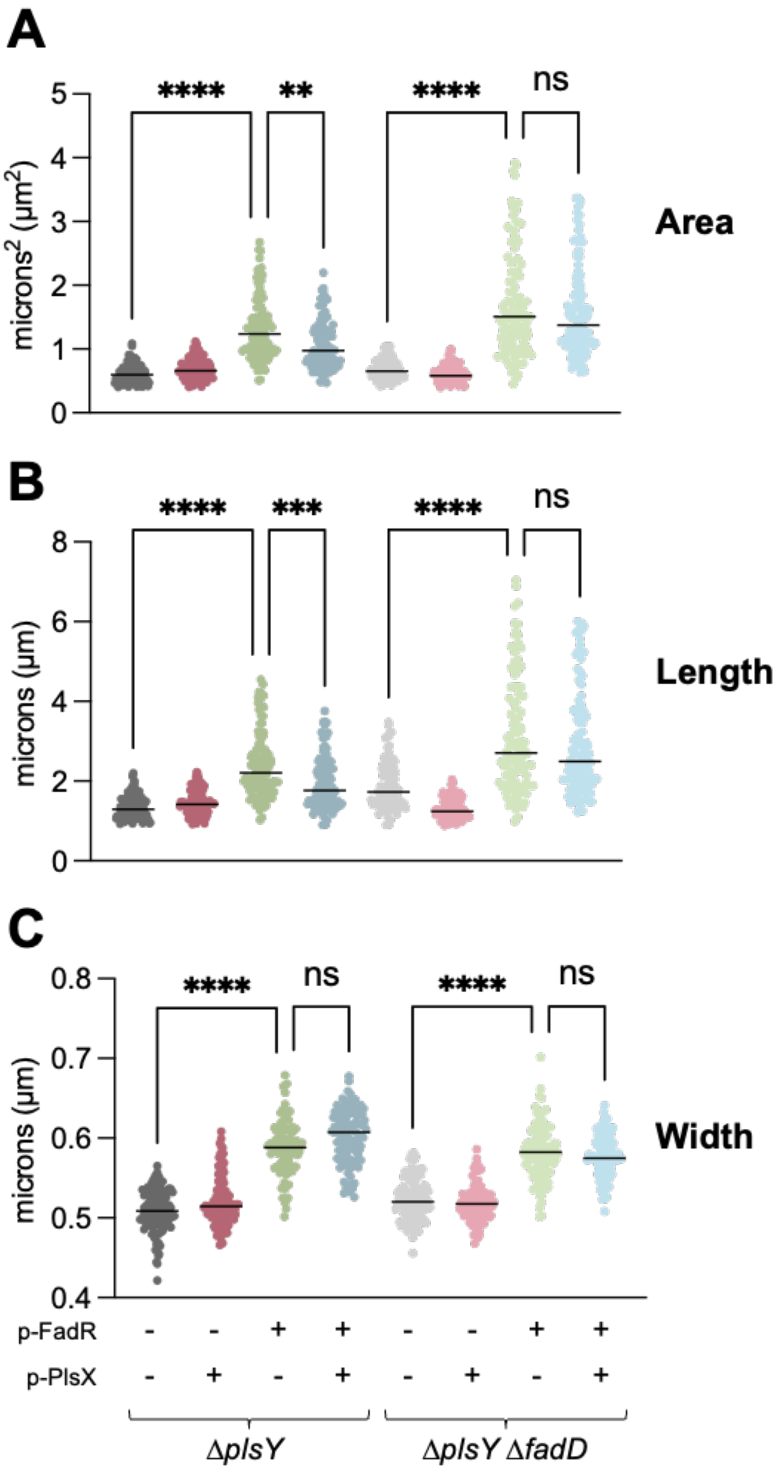

FIG S4 Overexpression of PlsX reduces increased cell size when FASII expression is upregulated. Summary analysis of phase contrast microscopy at 1000x magnification of Δ*plsY* and Δ*plsY* Δ*fadD* with overexpression of FadR and PlsX. Measurements of area (A), length (B), and width (C) are shown. Data represent mean values with the SD from >5 fields of view and >100 cells per strain. Significant was calculate using Brown-Forsythe and Welch ANOVA tests. **, $P \leq 0.01$; ***, $P \leq 0.0005$; ****, $P \leq 0.0001$; ns, not significant

Table S1. Co-transduction frequencies for determination of synthetic lethality in ∆*plsXY* and rescue with ∆*glpD*.

Parent Strain	P1 Phage	Expected Percent Co-Transduction	Frequency	Percent Co-Transduction
W3110	∆*plsY* ∆*ygjH::kan*	62.0%	41/80	51.25%
	∆*plsX* ∆*trhO::kan*	37.12%	10/84	11.90%
∆*plsX*	∆*plsY* ∆*ygjH::kan*	-	0/84	0.00%
∆*plsY*	∆*plsX* ∆*trhO::kan*	-	1/82*	0.01%
∆*plsX* ∆*glpD*	∆*plsY* ∆*ygjH::kan*	-	27/76	35.53%

Note: The one successful ∆*plsX* ∆*plsY* mutant generated (indicated by *) behaved phenotypically identical to suppressors described in Fig. 2.

DataSet S1 (separate file). Strains, plasmids, and primers used in this manuscript. Tab (1) Strains and plasmids used in this study. Strains that were assessed by whole genome sequencing are indicated. Tab (2) Primers used in this study**.** Tab (3) RNAseq data for Δ*plsX.*
